# Genome Sequence of the Quorum-Sensing-Signal-Producing Nonpathogen *Agrobacterium tumefaciens* Strain P4

**DOI:** 10.1128/genomeA.00798-13

**Published:** 2013-10-03

**Authors:** Samuel Mondy, Ouaghlis Lalouche, Yves Dessaux, Denis Faure

**Affiliations:** Institut des Sciences du Végétal, CNRS, Gif-sur-Yvette, France

## Abstract

*Agrobacterium tumefaciens* P4 is a quorum-sensing-signal-producing bacterium that has been isolated from the tobacco rhizosphere. This strain belongs to genomospecies 1 of the *A. tumefaciens* complex; it is avirulent on various putative host plants, devoid of the Ti plasmid, and contains a *luxI* homolog on the At plasmid.

## GENOME ANNOUNCEMENT

Among the cultured community collected from a tobacco rhizosphere, *Agrobacterium tumefaciens* strain P4 has been identified as an isolate that produces quorum-sensing (QS) signals of the *N*-acyl homoserine lactone (AHL) class (1). However, this strain is avirulent on different hosts (*Datura stramonium* and tomato plants) and defective for the plasmid Ti and, hence, for the *traI* gene that encodes the synthesis of the QS signal 3-oxo-octanoyl-homoserine lactone (3OC8-HSL) in agrobacteria. Using thin-layer chromatography, commercial 3OC8-HSL as a reference, and *A. tumefaciens* NT1(pZLR4) as an AHL biosensor strain (2), we confirmed that strain P4 produces a large amount of a molecule that is not 3OC8-HSL but indeed activates *Agrobacterium* QS-regulated *tra* genes. The exact structure of this molecule is currently being investigated.

Here, we report the *de novo* genome assembly of *A. tumefaciens* strain P4. Two libraries were constructed using the TruSeq SBS version 3 sequencing kit: a shotgun (SG) paired-end library with a fragment size between 150 and 500 bp and a long jumping distance (LJD) mate-pair library with an average insert size of 7,765 bp. The two libraries were sequenced using a 2 × 100 bp paired-end read module of Illumina HiSeq 2000 by Eurofins Genomics (France). Sequences reads with low quality (<0.05), ambiguous nucleotides (*n* > 1), and a sequence length of <50 nucleotides were discarded prior to assembly. After trimming, we retained 49,531,690 paired-end reads (4,695,604,212 bases) with an average length of 94.8 bp and 3,283,394 mate-paired reads (271,536,684 bases) with an average length of 82.7 bp. Sequence assembly was carried out using the CLC Genomics Workbench version 5.5 (CLC bio, Aarhus, Denmark), with a read length of 0.5 and a similarity of 0.8. Eighteen contigs were obtained with a length ranging from 2.4 kbp to 884 kbp, with an N_50_ value of 532,842 bp. The scaffolding was processed using SSPACE basic version 2.0 (3). The *in silico* finishing of some gaps was carried out by mapping (read length of 0.9 and similarity of 0.95) the mate-pair reads on each of the 5-kbp contig ends. Next, the collected reads were used for *de novo* local assembling (read length of 0.5 and similarity of 0.8). The published sequence is composed of nine contigs (from 53.8 kbp to 1.63 Mbp) grouped in 3 scaffolds, with a coverage rate ranging from 853- to 965-fold.

The *A. tumefaciens* strain P4 genome consists of one circular chromosome containing 2,856,286 bp, one linear chromosome containing 2,052,829 bp, and one circular At plasmid containing 661,825 bp. The G+C contents are 58.8%, 58.6%, and 56.7% for the circular chromosome, linear chromosome, and At plasmid, respectively. A total of 5,379 putative coding sequences were predicted using the Rapid Annotations using Subsystems Technology (RAST) version 4.0 automated pipeline (4). A survey of the P4 genome revealed the presence of a *luxI* homolog on the At plasmid, the function of which remains to be investigated.

### Nucleotide sequence accession numbers.

The *A. tumefaciens* P4 genome sequence has been deposited at DDBJ/EMBL/GenBank under the accession no. APJV00000000. The version described in this paper is the first version, APJV01000000.
